# Cost and Toxicity Comparisons of Two IMRT Techniques for Prostate Cancer: A Micro-Costing Study and Weighted Propensity Score Analysis Based on a Prospective Study

**DOI:** 10.3389/fonc.2021.781121

**Published:** 2022-01-11

**Authors:** Ingrid Masson, Martine Bellanger, Geneviève Perrocheau, Marc-André Mahé, David Azria, Pascal Pommier, Nathalie Mesgouez-Nebout, Philippe Giraud, Didier Peiffert, Bruno Chauvet, Philippe Dudouet, Naji Salem, Georges Noël, Jonathan Khalifa, Igor Latorzeff, Catherine Guérin-Charbonnel, Stéphane Supiot

**Affiliations:** ^1^ Department of Radiation Oncology, Institut de Cancérologie de l’Ouest René Gauducheau, Saint-Herblain, France; ^2^ Department of Human and Social Sciences, Institut de Cancérologie de l’Ouest René Gauducheau, Saint-Herblain, France; ^3^ UMR CNRS6051, EHESP (Ecole des Hautes Etudes en Santé Publique - School of Public Health), University of Rennes, Rennes, France; ^4^ Department of Radiation Oncology, François Baclesse Cancer Center, Caen, France; ^5^ Fédération Universitaire d’Oncologie Radiothérapie (FOROM), Institut Régional du Cancer Montpellier (ICM), Université de Montpellier, Institut de Recherche en Cancérologie de Montpellier (IRCM), Montpellier, France; ^6^ Department of Radiation Oncology, Léon Bérard Center, Lyon, France; ^7^ Department of Radiation Oncology, Institut de Cancérologie de l’Ouest Paul Papin, Angers, France; ^8^ Department of Radiation Oncology, Georges Pompidou European Hospital, Paris, France; ^9^ Department of Radiation Oncology, Lorraine Cancer Institute, Vandœuvre-lès-Nancy, France; ^10^ Department of Radiation Oncology, Sainte Catherine Institute, Avignon, France; ^11^ Department of Radiation Oncology, Pont de Chaume Clinic, Montauban, France; ^12^ Department of Radiation Oncology, Paoli-Calmettes Institute, Marseille, France; ^13^ Department of Radiation Oncology, Cancerology Institute of Strasbourg-Europe, Strasbourg, France; ^14^ Department of Radiation Oncology, Institut Universitaire du Cancer de Toulouse-Oncopole (IUCT-Oncopole), Toulouse, France; ^15^ Department of Radiation Oncology, Pasteur Clinic, Toulouse, France; ^16^ Clinical Trial Sponsor Unit/Biometry, Institut de Cancérologie de l’Ouest René Gauducheau, Saint-Herblain, France; ^17^ Centre de Recherche en Cancérologie et Immunologie Nantes Angers - Center for Research in Cancerology and Immunology Nantes-Angers (CRCINA), Institut National de la Santé et de la Recherche Médicale - National Institute for Health and Medical Research (INSERM) UMR1232, Centre National de la Recherche Scientifique - National Center for Scientific Research (CNRS) ERL6001, University of Nantes, Nantes, France

**Keywords:** Volumetric Arc Therapy (VMAT), helical tomotherapy (HT), high risk prostate cancers, micro-costing, inverse probability of treatment weighting (IPTW), toxicity, France

## Abstract

**Background:**

Intensity modulated radiation therapy (IMRT) combined with androgen deprivation therapy (ADT) has become the standard treatment for patients with high-risk prostate cancer. Two techniques of rotational IMRT are commonly used in this indication: Volumetric Modulated Arc Therapy (VMAT) and helical tomotherapy (HT). To the best of our knowledge, no study has compared their related costs and clinical effectiveness and/or toxicity in prostate cancer. We aimed to assess differences in costs and toxicity between VMAT and HT in patients with high-risk prostate cancer with pelvic irradiation.

**Material and Methods:**

We used data from the “RCMI pelvis” prospective multicenter study (NCT01325961) including 155 patients. We used a micro-costing methodology to identify cost differences between VMAT and HT. To assess the effects of the two techniques on total actual costs per patient and on toxicity we used stabilized inverse probability of treatment weighting.

**Results:**

The mean total cost for HT, €2019 3,069 (95% CI, 2,885–3,285) was significantly higher than the mean cost for VMAT €2019 2,544 (95% CI, 2,443–2,651) (p <.0001). The mean ± SD labor and accelerator cost for HT was €2880 (± 583) and €1978 (± 475) for VMAT, with 81 and 76% for accelerator, respectively. Acute GI and GU toxicity were more frequent in VMAT than in HT (p = .021 and p = .042, respectively). Late toxicity no longer differed between the two groups up to 24 months after completion of treatment.

**Conclusion:**

Use of VMAT was associated with lower costs for IMRT planning and treatment than HT. Similar stabilized long-term toxicity was reported in both groups after higher acute GI and GU toxicity in VMAT. The estimates provided can benefit future modeling work like cost-effectiveness analysis.

## Introduction

Intensity modulated radiation therapy (IMRT) combined with androgen deprivation therapy (ADT) for 2–3 years has become the standard treatment for patients with high-risk prostate cancer (1–6). The further technological improvement with rotational modulated radiotherapy makes it possible to achieve dose escalation in the primary tumor while sparing normal tissues or organs. Two techniques of rotational IMRT are commonly used: Volumetric Modulated Arc Therapy (VMAT RapidArc™ Varian Medical Systems and VMAT^®^ Elekta) and helical tomotherapy (HT: Tomotherapy^®^ Accuray).

Several studies have suggested more favorable dosimetric parameters with VMAT when compared with step-and-shoot or conventional IMRT in patients with prostate cancers, including high-risk prostate cancers (7–12). VMAT results in improved sparing of organs at risk (OARs) and delivery efficiency, combined with significantly lower treatment time and reduced monitor units (MUs) (7–12). There was no worsening clinical effectiveness or toxicity reported (7).

When comparing VMAT techniques and helical tomotherapy (HT) only small significant dosimetric differences in prostate cancer were observed (13) including high-risk prostate cancer with pelvic nodal irradiation (14). Both accelerators exhibited the same plan quality (13), but with more homogeneous dose distribution for HT (14) and in some studies, better OAR sparing for HT (15, 16), whereas VMAT was associated with shorter treatment time and lower MUs (13, 14, 16). There was no comparative analysis of clinical effectiveness or toxicity of VMAT versus HT in high-risk prostate cancer, whether the radiotherapy was to the prostate alone or to the prostate and pelvic lymph nodes.

Among attempts to estimate costs and effectiveness associated with these advanced technologies, we retrieved only one in prostate cancer that compared VMAT to IMRT and found it “cost-effective” (7). In a recent systematic review on cost-effectiveness of prostate cancer radiotherapy, stereotactic body radiotherapy (SBRT) appeared the least expensive from a societal perspective followed by IMRT. Proton beam therapy (PBT) was both the most expensive and the least effective (17). Nevertheless, lack of evidence in cost estimates and “uncertainty surrounding improvements in outcomes” of new technologies (e.g., disease progression, adverse events) make cost-effectiveness analysis of “new versus traditional technologies of radiotherapy” in prostate cancer challenging (18).

To the best of our knowledge, no study has compared costs and clinical effectiveness and/or toxicity of VMAT versus HT in prostate cancer. Only two studies (19, 20) from a French prospective multicenter study evaluated the clinical outcomes and costs associated with these techniques in patients with head and neck cancer.

The present study aimed to assess differences in costs and toxicity between VMAT and helical tomotherapy in patients with high-risk prostate cancer. For this study, we considered no differences between VMAT RapidArc™ and VMAT^®^ Elekta, as done in the literature, since this is the same VMAT technology developed by two manufacturers. We referred to them both as VMAT, when appropriate.

## Materials and Methods

### Study Design

We used data from the “Intensity modulated Radiation Therapy in pelvic lymph node irradiation” prospective multicenter study (*so called in French “RCMI pelvis”)*, which aimed to compare costs and clinical outcomes of IMRT with VMAT (Rapid’Arc™ and VMAT^®^ Elekta) versus helical tomotherapy (HT) (Tomotherapy^®^) in prostate, cervical, and anal canal cancers, with pelvic lymph node irradiation (https://www.clinicaltrials.gov/, NCT01325961). The “RCMI pelvis” study had the same protocol as the ‘ART-ORL’ study on head and neck cancer, and both studies were carried out jointly by fourteen French academic cancer centers (**Supplementary Table A1**) (19, 20). Patients were assigned to one of the accelerators based on their availability in each center or at the discretion of the investigators. No randomization between treatments was thus possible. The expected number of patients to be included was 20 anal canal cancers, 50 cervical cancers, and 150 prostate cancers. However, to obtain a homogenous population, for the current study we only selected patients with prostate cancer. The inclusion criteria were patients aged ≥18 years with histologically proven high-risk prostate cancer according to d’Amico’s classification (21) and the Eastern Cooperative Oncology Group (ECOG) Performance Status ≤2, and who received pelvic lymph node irradiation and androgen deprivation therapy (ADT) for 3 years. Patients included in the GETUG 18 trial were also eligible for the RCMI pelvis study (22). The exclusion criteria were lombo-aortic irradiation, salvage radiotherapy after radical prostatectomy, and re-irradiation. Enrolled patients had an abdominal, pelvic, and thoracic CT Scan and/or Positron Emission Tomography–Computed Tomography (PET-CT) and Prostate specific antigen (PSA) test. Delineation followed guidelines for high-risk prostate cancer (22). Clinical Target Volume (CTV) 1 included pelvic lymph node areas, prostate, and seminal vesicles. CTV 2 was limited to the prostate. Planning Target Volume (PTV) 1 and 2 were respectively defined by: CTV1 + 1 cm, CTV2 + 1 cm (+0.5 cm posterior). A moderate hypofractionated Simultaneous Integrated Boost (SIB) plan was delivered to 34 fractions: 54.4 Gy to PTV1 and 74.8 Gy to PTV2. For GETUG 18 trial patients only, a normo-fractionated sequential plan was delivered in 40 fractions: 46 Gy to PTV 1 then 34 Gy to PTV2. Dose prescription followed the International Commission on Radiation Units 83 (23). All patients provided signed informed consent to participate in the study. The study was approved by the National Ethics Committee (Ref: 11/03, 4 January 2011) and the National Committee for protection of personal data (DR-2011-277 N°911317).

### Micro-Costing Study

The micro-costing methodology is considered the most accurate approach for costing hospital services (24). It makes it possible to identify all the resources used within hospital production processes and per patient (24). In our micro-costing study, we defined the production process as being from treatment planning to the last radiation therapy (RT) session. Of note, ADT was not included in the costing analysis, since this is a standardized procedure. To measure and value the resources used, we followed, and adapted when necessary, the previously described method of Perrier et al. (19). For this reason, we only identified use of resources that was likely to vary between VMAT and HT to estimate actual cost differences between the two strategies. Our study assumed differences in the resources used in all planning stages (i.e., image registration and contouring, inverse planning, patient-specific quality control (QC), and pre-treatment patient setup verification), along with treatment delivery, accelerator QC for IMRT, and preventive internal and external maintenance. CT scan planning was supposed to be similar in both techniques. We did not include Record and Verify Systems (RVSs) as Tomotherapy treatments were already integrated into the RVSs from the other manufacturers. Patient-specific Quality Assurance (QA) software, independent of the machine, was excluded accordingly. Last, we considered standard radiation bunker that may accommodate any accelerator currently used (19) and also any change in radiotherapy advancements over its life span. We therefore assumed similar construction costs for the two techniques studied.

Detailed data on resource utilization of labor and equipment were collected using chronometers. They included all personnel time spent and equipment mobilization time for each patient over their course of treatment. For example, for a treatment session, this ranged from a patient’s admission to discharge from the irradiation room. We thus included not only the time of treatment delivery, but also image control time and any potential interruption time. Labor included radiation therapist, dosimetrist, medical physicist, radiation oncologist, resident and biomedical technician. We assigned values of time resources used to estimate costs, as follows. Unit costs of labor were based on the average annual full gross wages (i.e., annual payroll taxes included) divided by the number of workable yearly hours. We estimated the full gross wage on a 15-year experience basis and on a collective labor agreement of UNICANCER, a consortium of the 18 French cancer centers. For all cost estimates, we used the same method as described in Perrier et al. (19), except for the costs of QC and internal maintenance (IM), given in Equation (1) below and for which resources were collected from questionnaires.


(1) $$\text{QC and IM cost}=\Sigma_{\text{labor}=1}^{2}\text{ hourly full wage}*\text{QC and IM Nhs}$$

Where Nhs = annual number of dedicated hours, and labor = medical physicist or biomedical technician.

We obtained data on capital resources, such as equipment (i.e., accelerator and Treatment Planning Systems—TPS—for VMAT only, as for HT accelerator cost includes TPS) from standardized questionnaires, as previously described (19). Equipment was valued using replacement and maintenance costs with a life expectancy of 12 years for the accelerators and 5 years for the TPS, based on data available from the literature (25, 26). We estimated the costs of accelerators, annual external maintenance contracts and TPS directly from the catalog prices of the manufacturers.

We estimated total actual costs per patient that vary between VMAT and HT from the hospital perspective. All estimates were in Euro 2019, all taxes included, but without discounting as the analytic time horizon for costs was less than 3 months. It is worth noting that we could not estimate the resources used for treatments associated with acute toxicity, since all treatment types, but hospitalization, were not collected in the frame of the study.

### Toxicity

We analyzed acute toxicity (≤3 months) and late toxicity [up to 24 months, based on previously published studies (27, 28)], that were scored using the National Cancer Institute Common Terminology Criteria for Adverse Events (CTCAE) version 3.0. Toxicity was reported as follows: no toxicity (grade 0), grade 1, grade 2, and grades 3–4. Acute adverse events (serious adverse events with hospitalizations included) that were clinical outcomes within the same time horizon as the estimated costs were reported on electronic Case report forms (CRF). Besides, all late adverse events were collected as well. For the analysis, we reported Gastrointestinal (GI) toxicity, Genito-urinary (GU) toxicity, and sexual toxicity. Lastly, patient reported outcomes such as Quality of Life (QoL) were not investigated in this study.

### Statistical Analysis

We described patients’ characteristics using mean and standard deviation (SD) for continuous variables, or count and percentage for categorical variables. In addition, we assessed differences between treatment groups, from the original datasets, using logistic regression, and multinomial logistic regression for binary variables and tumor stage (cT) respectively, and linear regression for continuous variables. We compared differences in hours spent for treatment, QC, and internal maintenance between the two techniques using a non-parametric Wilcoxon test and we used a paired Wilcoxon test for comparing differences in duration of treatment session. We tested uncertainty on the total cost of each technique by running a one-way sensitivity analysis over the range of plausible parameters and illustrating with a Tornado plot showing the impact of increasing and decreasing each of the parameters by 20%, a range suggested in the methods of sensitivity analysis in economic evaluation (29). In addition, we used probabilistic sensitivity analyses with a non-parametric bootstrap method, with 1,000 iterations, to obtain the 95% confidence intervals (CI) of total actual costs (30).

Similarly to Bibault et al. (20), we used propensity scores to obtain balancing covariates at enrollment in the VMAT and HT groups to address potential selection bias due to non-randomization (31). We used the stabilized inverse probability of treatment weighting (IPTW) (32). We estimated standardized differences to compare the balance in measured baseline covariates between the two treatment groups. Due to the study design, we could not include cancer center as a covariate, though it was likely to be a confounding factor. As mentioned above, investigators assigned patients to one of the accelerators based on their availability in each cancer center or at their discretion. We estimated the propensity score (PS) using a logistic regression in which treatment assignment was regressed (32). With ITPW, each patient was weighted by the inverse probability of receiving the treatment they actually received (i.e., 1/PS in the HT group and 1/(1 − PS) in the VMAT group). Finally, we applied the stabilized IPTW, to preserve the size of the pseudo-population as well as to reduce the variance of the treatment effect estimates (32).

To assess the impact of the two techniques on total costs and toxicity we ran linear regressions (costs) and ordered logistic regressions (toxicity) adjusting for sequential versus SIB plans. All regressions were performed before and after having introduced IPTW.

We performed all analyses using R software version 3.6.1. We used the stdidff package (https://CRAN.R-project.org/package=stddiff) to compute the standardized differences for all categorical variables with more than two levels.

## Results

### Patient Characteristics

Two hundred and fifteen patients were recruited between April 2011 and January 2015: 155 with prostate cancer, 30 with cervix cancer and 30 with anal cancer (**Supplementary Figure A1**). Of the 155 patients with prostate cancer, 106 patients were treated with VMAT and 49 with HT. We had information on resource use for all patients (155); and for toxicity, information was available for 147 patients (i.e., 98/106 in the VMAT group; 49/49 in the HT group).

At baseline, age, performance status, cT and cN stages, and PSA varied between the two techniques, (with significant differences for N stage). After IPTW, we observed almost no differences between the VMAT and HT groups (**Table 1**). For the Gleason scores, when checking the balance between groups we found no significant difference before or after weighting (p >0.5).

**Table 1:** Patient characteristics.

	Unweighted				Weighted		
	VMAT (n = 106)	HT (n = 49)	P-value	d	VMAT (Pseudo-data)	HT (Pseudo-data)	d
**Age (years)**			.3084	0.171			−0.002
mean (SD)	68 (7)	70 (9)			69 (7)	69 (9)	
**Performance status**			.1078	0.271			−0.010
0	93 (87.7%)	38 (77.6%)			84.7%	85.1%	
1 or 2	13 (12.3%)	11 (22.4%)			15.3%	14.9%	
**cT stage**				0.356			0.048
cT1	14 (13.2%)	9 (18.4%)			15.0%	15.9%	
cT2	35 (33.0%)	10 (20.4%)	.1460		28.2%	26.2%	
cT3	55 (51.9%)	27 (55.1%)	.5802		53.7%	54.6%	
cT4	2 (1.9%)	3 (6.1%)	.4005		3.1%	3.3%	
**N stage**			.0322	0.361			0.002
cN0	96 (90.6%)	38 (77.6%)			85.5%	85.5%	
cN1	10 (9.4%)	11 (22.4%)			14.5%	14.5%	
**PSA (ng/ml); capped values**			.2652	0.194			0.024
mean (SD)	15 (13)	18 (13)			17 (14)	17 (12)	

d, Standardized difference; IPTW, Inverse probability of treatment weighting; HT, helical tomotherapy.

### Resource Use

We observed similar median annual treatment times (Interquartile range—IQR) spent with VMAT and helical tomotherapy (HT), with 2,513 h (IQR, 92.5) and 2,520 h (IQR, 216), respectively (P = .7). In addition, we did not find any statistical differences in median annual time spent for quality control and internal preventive maintenance, 194.5 h (IQR, 81.75) were spent with VMAT and 144 h (IQR, 56) with HT (p = .28). We found that the median time for the first session was 18 min per patient for HT and 20 min for VMAT (p = .31) (**Supplementary Figure A2**). Treatment times then decreased across sessions for VMAT (15 min) while keeping the same for HT (18 min) (p = .002 and p <.001), up to the fourth and more sessions, for which 13 and 17 min (p <.001) were reported for VMAT and HT, respectively. This demonstrates a learning process effect on time spent per patient.

### Total Actual Costs per Patient

**Table 2** presents the total actual costs per patient as well as major cost components. After inverse probability of treatment weighting and control for covariates, the mean cost for helical tomotherapy (HT), estimated at € 3,069 (95% CI, 2,885–3,285) was significantly higher than the mean cost for VMAT €2,544 (95% CI, 2,443–2,651) (p <.0001). We found a substantial variation in session costs (i.e., accelerator + labor costs), accounting for 94% of HT costs and only 78% of VMAT costs (p <.001) (**Table 2**). As shown in **Table 2**, only the planning phase was more costly for VMAT than for HT, with €566 (292), and €189 (117) mean costs (SD), respectively (p <.0001) (**Table 2**). This yielded a mean cost difference of €525 in the HT group compared with the VMAT group.

**Table 2:** Total actual cost per patient (€2019) after Inverse probability of treatment weighting (IPTW).

Cost	VMAT	HT	P-value
	mean (SD)	mean (SD)	
Image registration, contouring—Labor	109.10 (76.47)	38.75 (56.59)	–
Image registration, contouring—TPS	95.98 (65.79)	0.00 (0.00)	–
Image registration, contouring—Total	205.07 (139.93)	38.75 (56.59)	<.0001
Inverse planning and validation—Labor	141.47 (104.02)	67.49 (66.39)	–
Inverse planning and validation—TPS	133.48 (90.17)	0.00 (0.00)	–
Inverse planning and validation—Total	274.96 (191.66)	67.49 (66.39)	<.0001
Patient quality control—Labor	19.96 (12.12)	24.00 (10.43)	.0574
Position verification D0*—Labor	22.94 (30.40)	12.21 (7.66)	.0272
Setup verification D0* Accelerator	43.27 (56.24)	46.19 (25.85)	.6200
Setup verification D0*—Total	66.21 (84.83)	58.40 (32.78)	.6529
** Planning Cost**	**566.19 (292.71)**	**188.64 (116.58)**	**<.0001**
Session—Labor	484.52 (135.28)	546.84 (120.39)	.0050
Session—Accelerator	1,493.66 (362.39)	2,333.30 (466.52)	<.0001
** Session Cost**	**1,978.18 (475.14)**	**2,880.13 (582.64)**	**<.0001**
** Total actual cost** CI 95%**	**2,544.37** **[2,442.88; 2,651.11].**	**3,068.77** **[2,885.34; 3,285.04]**	**<.0001**

*D0 pre-treatment patient setup verification.

**95% confidence intervals (CI) of total actual costs computed based on probabilistic sensitivy analyses with a non-parametric bootstrap method, with 1,000 iterations excluding (25/1,000) 2.5% values at either end of the estimated distribution (30).

HT, helical tomotherapy.

**Figure 1:**
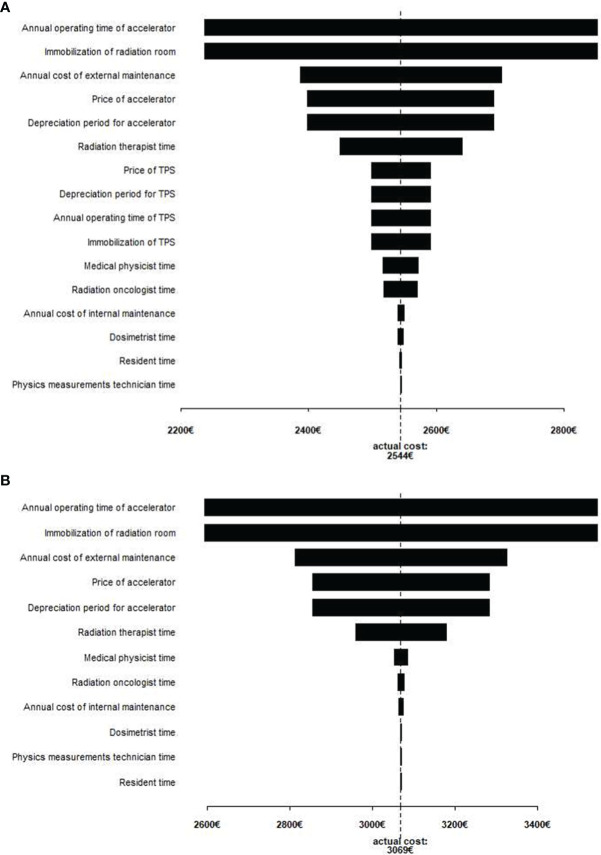
Tornado diagram for VMAT **(A)** and helical tomotherapy **(B)** total actual costs. Tornado diagram shows the graphical output of the one- way sensitivity analysis. On the x-axis, the value of total cost, and the vertical line represents the total cost with all parameter baseline values. Each parameter has its own bar and the length of each bar shows how much impact that parameter can have total cost when varied 20% more of less than its baseline value is. The bars are arranged in descending order of length, so that the diagram exhibits from the most to the least sensitive factors.

The Tornado diagram (**Figure 1**) shows the most influential parameters (i.e., prices, cost, time with ±20% variation) on estimated costs. The latter were highly sensitive to the annual operating time as well as to the accelerator immobilization time (**Figure 1**). The costs decreased by 12.1 and 15.5% following a 20% increase in the annual operating time and a 20% decrease in the accelerator immobilization time, for VMAT and for HT, respectively. To a lesser extent, costs were sensitive to time spent by the radiation therapist (**Figure 1**).

For each technique, the sequential plan exhibited significantly higher costs than the SIB plan (p <.001), with €2,659 (95% CI, 2,504–2,834) versus €2,498 (95% CI, 2376–2657) for VMAT, and €3,902 (95% CI, 3473–4357) versus €2,895 (95% CI, 2754–3076) for helical tomotherapy. The variation was mainly due to the irradiation phase, where accelerator immobilization time and labor time were more expensive in the sequential process than in the SIB process (**Supplementary Figure A3**).

### Acute and Late Toxicity

As shown in **Table 3A**, the proportion of patients with acute grade 3–4 toxicities was very low in both groups. This went from 0 to 1% of patients having Gastrointestinal (GI) toxicity (p = .033) to 4% with genitourinary (GU) toxicity in both groups (p = .145). After IPTW and control for covariates, we observed significantly higher frequency in acute GI and GU toxicities for VMAT than for HT (p = .021 and p = .042, respectively) but similar sexual toxicities in the two groups (p = .236).

**Table 3:** Acute and late toxicity.

TABLE 3A. Acute Toxicity*							
Toxicity	Grade*	Unweighted			Weighted		
		VMAT (n = 98)	HT (n = 49)	P-value	VMAT	HT	P-value
GI toxicity	0	17 (17.3%)	14 (28.6%)	**.0339**	16.6%	29.2%	**.0215**
	1	48 (49.0%)	26 (53.1%)		47.3%	51.6%	
	2	32 (32.7%)	9 (18.4%)		34.9%	19.3%	
	3–4	1 (1.0%)	0 (0.0%)		1.2%	0.0%	
GU toxicity	0	10 (10.2%)	7 (14.3%)	.1446	9.5%	16.1%	**.0420**
	1	34 (34.7%)	24 (49.0%)		34.5%	50.9%	
	2	50 (51.0%)	16 (32.7%)		52.2%	29.7%	
	3–4	4 (4.1%)	2 (4.1%)		3.9%	3.3%	
Sexual toxicity	0	49 (50.0%)	33 (67.3%)	.2628	48.6%	66.2%	.2369
	1	21 (21.4%)	1 (2.0%)		21.5%	3.4%	
	2	27 (27.6%)	15 (30.6%)		28.7%	30.3%	
	3–4	1 (1.0%)	0 (0.0%)		1.2%	0.0%	

GI, gastro-intestinal; GU, genito-urinary; HT, helical tomotherapy.

*Acute toxicity: worse grade during three months of follow up.

**Table 3b:** Late toxicity*.

Toxicity	Grade*	Unweighted			Weighted		
		VMAT (n = 98)	HT (n = 49)	P-value	VMAT	HT	P-value
GI toxicity	0	41 (41.8%)	27 (55.1%)	.2964	41.4%	59.2%	.1627
	1	38 (38.8%)	13 (26.5%)		39.3%	21.4%	
	2	18 (18.4%)	7 (14.3%)		18.4%	16.2%	
	3-4	1 (1.0%)	2 (4.1%)		0.9%	3.2%	
GU toxicity	0	26 (26.5%)	14 (28.6%)	.4797	25.3%	35.3%	.6696
	1	50 (51.0%)	20 (40.8%)		49.7%	38.4%	
	2	20 (20.4%)	14 (28.6%)		23.2%	23.3%	
	3-4	2 (2.0%)	1 (2.0%)		1.8%	3.0%	
Sexual toxicity	0	38 (38.8%)	15 (30.6%)	**.0214**	37.7%	32.0%	.0628
	1	19 (19.4%)	1 (2.0%)		19.1%	3.7%	
	2	37 (37.8%)	33 (67.3%)		39.2%	64.3%	
	3-4	4 (4.1%)	0 (0.0%)		4.0%	0.0%	

GI, gastro-intestinal; GU, genito-urinary; HT, helical tomotherapy.

*Late toxicity: worse grade from 6 months onwards.

In the long-term period, in both unweighted and weighted populations, the proportions of patients for whom physicians reported late grade 3–4 toxicities remained dramatically low (with no more than 4% of patients experiencing grade 3–4 GI, GU and sexual toxicity), whatever the machine (**Table 3B**). Overall, VMAT and HT did not differ significantly in late GI, GU and sexual toxicity either (p = .162; p = .669; and p = .062 respectively after IPTW) (**Table 3B**).

There was no difference in serious adverse event related hospitalization, nor any permanent discontinuation of treatment reported in the study. In acute toxicity, we observed only one hospitalization due to dysuria in the HT group. In late toxicity, three hospitalizations were noted for dysuria on stenosis, hematuria, and rectal bleeding on radiation proctitis in the VMAT group and one for rectal bleeding on radiation proctitis in the HT group.

## Discussion

The analysis based on the “RCMI pelvis” prospective multicenter study found that each hospital spent on average an additional €525 per patient when they used helical tomotherapy (HT) rather than VMAT for IMRT preparation and delivery in high-risk prostate cancer patients with pelvic lymph node irradiation. Considering health outcomes, although acute GI and GU toxicity were significantly higher for VMAT than for HT, the late toxicity outcomes (up to 24 months after completion of treatment) no longer differed between the two groups.

The economic results are consistent with the data on head and neck cancers published by Perrier et al., which show an excess in mean costs for HT over VMAT (19). In the two studies, the findings were explained by differences in the price of accelerators and in costs of external maintenance, along with differences in treatment times. Helical tomotherapy has remained more expensive than VMAT accelerators, despite a reduction in price variation between 2013 and 2019, the two years of estimation. In addition, longer RT sessions result in increased times spent by personnel and accelerators. The results from our study are also consistent with existing evidence on treatment delivery as a major cost component of IMRT (33–35). In line with our estimates, and for prostate cancer, HT was associated with a longer average treatment delivery time than VMAT (15, 36). Of note, this finding holds true, whether time was measured only for irradiation (15, 36) or for the whole treatment delivery as we and Perrier et al. (19) did. For VMAT mostly, we observed a learning effect, which was previously demonstrated during the implementation process of new techniques (37). The micro-costing methodology makes possible time assessment that is closest to clinical practice. However, our evaluation differs from Perrier et al., as we estimated all times spent (such as operating time, QC time of treatment machines and internal maintenance) from standardized questionnaires completed by the cancer centers included in the study. This resulted in findings in line with the Health Economics in Radiation (ESTRO/HERO) project for which annual QC time estimated was 150 to 200 h, based on a time-driven, activity-based, costing methodology (TD-ABC) (25). Finally, the study observed lower costs for SIB hypofractionated IMRT compared to sequential IMRT in prostate cancers, and this, regardless of the accelerators, as in Hulstaert et al. who found lower average costs in hypofractionated schemes in lung and breast cancers compared to standard fractionation schemes (35). In the latter, treatment delivery was the major cost driver, a factor of cost variation that we also observed in the sensitivity analysis we carried out.

In our micro-costing study, we were able to refer to the Diagnosis-Related Group (DRG) related tariff for a given procedure, and that the national health insurance system (NHIS) reimburses hospitals. As reported in a recent European study, a higher reimbursement is made in France for treatments with helical tomotherapy when comparing to other VMAT machines (38). The current tariff for a HT session is twice that of a VMAT session when our actual estimations showed a 20% incremental cost (i.e., a € 525 (95% CI, 442–634) difference between the two techniques).

In terms of toxicity, comparative evidence between VMAT and helical tomotherapy is limited. Our study found significant higher frequency in acute GI and GU in VMAT than in HT, but no worse significant outcomes in late toxicity. Our results are similar to those of Bibault et al. in head and neck cancers (20), who after IPTW adjustment found significantly more acute salivary disorders in the VMAT group, but no difference between HT and VMAT in all toxicities evaluated in the long term (more than 15), except for salivary function (20). Stabilized long term symptoms after significant differences in acute symptoms associated with IMRT have been reported elsewhere (39).

### Strengths and Limitations

This study used rigorous methods to assess the costs and toxicity associated with helical tomotherapy (HT) and VMAT IMRT in prostate cancers. The large variation in baseline characteristics between the two techniques demonstrated the importance of using the propensity score as it makes appropriate adjustment possible for reducing confounding bias when estimating treatment effects. In the economic analysis of radiotherapy, micro-costing represents an accurate method for collecting details of the resources used for costing procedures. To our knowledge, this study is the first of its kind to estimate the costs and toxicity of VMAT and HT in high-risk prostate cancers with pelvic lymph node irradiation with long follow-up time. The identification of treatment delivery as a major cost driver and source of difference between the techniques used is a result of particular interest, given cancer centers’ efforts to introduce new radiotherapy techniques. Applying micro-costing methodology contributes to a broader European (EU) effort to improve knowledge of the resources allocated to radiotherapy within the overall resources for treating cancer patients (26, 38, 40–43), and ultimately to improve the quality of care. However, one should keep in mind that our evaluation was based on French cancer center labor costs and payment schemes, which differ from other EU and international oncology centers (38). This should be considered carefully before generalizing our findings.

This study has certain limitations. The first relate to the data collection. Collecting data in real-world clinical practice has some advantages, but it can also be challenging on both the clinical and the funding sides. This has resulted in variability in patients recruited across cancer centers and in a small population sample. In addition, randomization between treatment groups was not possible. We note that the propensity scores we used do not account for the effect of unmeasured covariates (44, 45). Because patients were assigned to only one technique in each cancer center, based on their availability (only 4 centers out of 14 had both VMAT and HT accelerators), or on investigators’ discretion, as earlier mentioned, we could not take the cancer center effect into account, although we expect that it is a confounding factor. Another limitation of the study is the difference in time horizon between the cost analysis and the toxicity assessment, due to data collected. We were not able to estimate the costs of treatments used for acute adverse events. This has resulted in underestimating short term costs, and has made impossible to relate the differences in cost to the differences in outcomes, a requirement in cost-effectiveness analysis. Moreover, as in any economic evaluation, and especially when conducting a micro-costing study, we made assumptions and simplifications that Perrier et al. have already reported (19) and that may have an impact on our final estimates. We thus advise caution before generalizing our results. Finally, our model does not apply to low- or intermediate risk prostate cancer patients where pelvic irradiation is not routinely recommended (46), where ADT is usually limited to unfavorable intermediate-risk tumors (5), and SBRT or proton therapy to the prostate gland only is a therapeutic option (46).

## Conclusion

Assessing the effectiveness and value associated with the IMRT treatment of prostate cancer with pelvic lymph node irradiation is of particular importance. The study found evidence to support the hypothesis of cost differences between VMAT and HT for IMRT preparation and delivery in favor of VMAT, and no toxicity differences in the long term after more acute GI and GU toxicity for VMAT than for HT. Our findings have already the potential to help clinicians’ decision-making. For the payer, the French NIHS, these findings support an increase in the DRG reimbursement of a VMAT session. Our approach paved the way to a cost-effectiveness model that will combine the long-term health impacts of the two techniques, already identified, to the quality of life reported by patients to be assessed along with long-term costs of VMAT and HT.

## Data Availability Statement

The data that support the findings of this study are available on request from the last author (SS).

## Ethics Statement

The study was approved by the National Ethics Committee (Ref: 11/03, 4 January 2011) and the National Committee for protection of personal data (DR-2011-277 N°911317). The patients/participants provided their written informed consent to participate in this study.

## Author Contributions

Conceptualization, M-AM and GP. Methodology, IM, MB, GP, CG-C, and SS. Software, MB and CG-C. Validation, IM, MB, GP, CG-C, and SS. Formal analysis, IM, MB, GP, and SS. Investigation, DA, PP, NM-N, PG, DP, BC, PD, NS, GN, JK, IL, and SS. Resources, IM, MB, and GP. Data curation, IM, GP, and CG-C. Writing—Original draft preparation, IM, MB, and SS. Writing—Review and editing, IM, MB, and SS. Visualization, IM, MB, and SS. Supervision, MB and SS. Project administration, GP. Funding acquisition, M-AM. All authors contributed to the article and approved the submitted version.

## Funding

The three manufacturers (Accuray, Inc., Varian Medical Systems, and Elekta AB) financially supported the RCMI pelvis study. However, the RCMI pelvis is an independent study and the ideas and opinions expressed in this work are those of the authors and do not necessarily represent those of the manufacturers. The funders (Accuray, Inc.; Varian Medical Systems; Elekta AB) had no role in the design of the study; in the collection, analyses, or interpretation of data; in the writing of the manuscript, or in the decision to publish the results.

## Conflict of Interest

DA declares conflict of interest with Novagray, which has nothing to do with this study.

The remaining authors declare that the research was conducted in the absence of any commercial or financial relationships that could be construed as a potential conflict of interest.

## Publisher’s Note

All claims expressed in this article are solely those of the authors and do not necessarily represent those of their affiliated organizations, or those of the publisher, the editors and the reviewers. Any product that may be evaluated in this article, or claim that may be made by its manufacturer, is not guaranteed or endorsed by the publisher.
