# Supplementary material for: Cost and Toxicity Comparisons of Two IMRT Techniques for Prostate Cancer: A Micro-Costing Study and Weighted Propensity Score Analysis Based on a Prospective Study
Source: Front Oncol. 2022 Jan 11;11:781121. doi: 10.3389/fonc.2021.781121 (PMC8787862; doi:10.3389/fonc.2021.781121)
Supplement: Supplementary file 1 [file DataSheet_1.docx]

**Appendix**

**Table A1**. List of the 14 centers that enrolled patients for the RCMI pelvis study

| **Type** | **Center** | **City** |
| --- | --- | --- |
| **Academic** | René Gauducheau Cancer Institute | Saint-Herblain |
|  | Montpellier Cancer Institute | Montpellier |
|  | Lorraine Cancer Institute | Vandoeuvre-lès-Nancy |
|  | Léon Bérard Center | Lyon |
|  | Paul Papin Cancer Institute | Angers |
|  | Georges Pompidou European Hospital | Paris |
|  | Paul Strauss Cancer Institute | Strasbourg |
|  | Paoli-Calmettes Institute | Marseille |
|  | IUCT Oncopole | Toulouse |
|  | Bergonié Institute | Bordeaux |
|  | Curie Institute | Paris |
| **Private for**  **profit** | Sainte Catherine Institute | Avignon |
|  | Pont de Chaume Clinic | Montauban |
|  | Pasteur Clinic | Toulouse |

**Included in RCMI pelvis**

**(n = 215)**

**Excluded (n= 60)**

Cervical cancers (n=30)

Anal canal cancers (n=30)

Anal canal cancers (n=30)

**Duration of inclusion = 3 years & 10 months**

**Eligible for prostate cancer analysis (n=155) including**

- Return from default (RfD): n =3

- Withdrawal of consent (WoC) after 12 months of follow-up: n=1

- Minor deviations (MD) to protocol: n=14 (imaging not performed at inclusion)

**HT**

**n = 49**

**VMAT**

**(Rapid’Arc™ and VMAT® Elekta)**

**n = 106** including

- RfD : n=3
- WoC: n=1
- MD : n=14

**Figure A1.** Patient flow chart

Abbreviations: RfD = Return from default; WoC = Withdrawal of consent; MD = Minor deviations; HT = helical tomotherapy


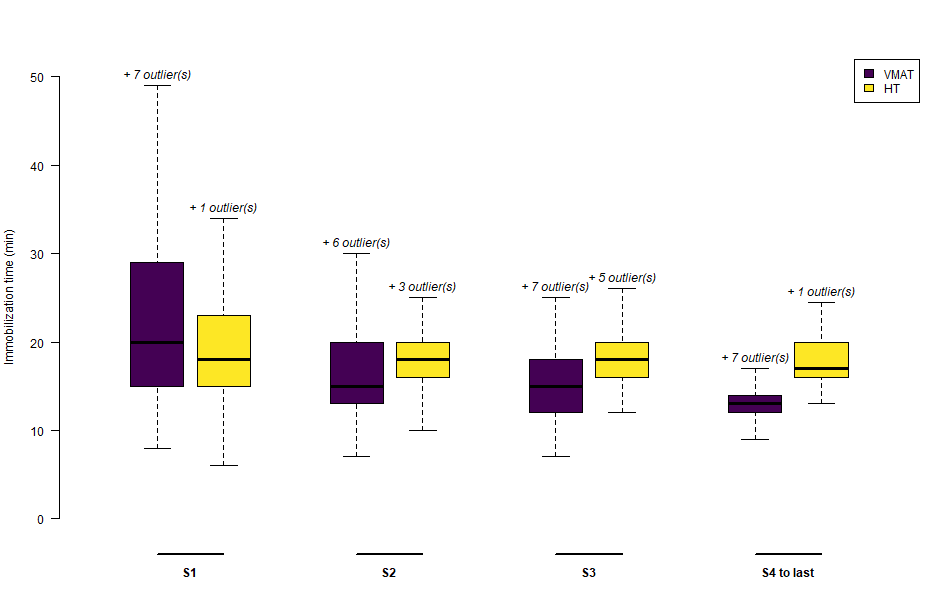


**Figure A2.** Comparison of session times between VMAT and helical tomotherapy.

The results are presented separately for sessions 1, 2 and 3 and then the results have been pooled from session 4 to the last session of radiation therapy.

Abbreviation: S = session, HT = helical tomotherapy.


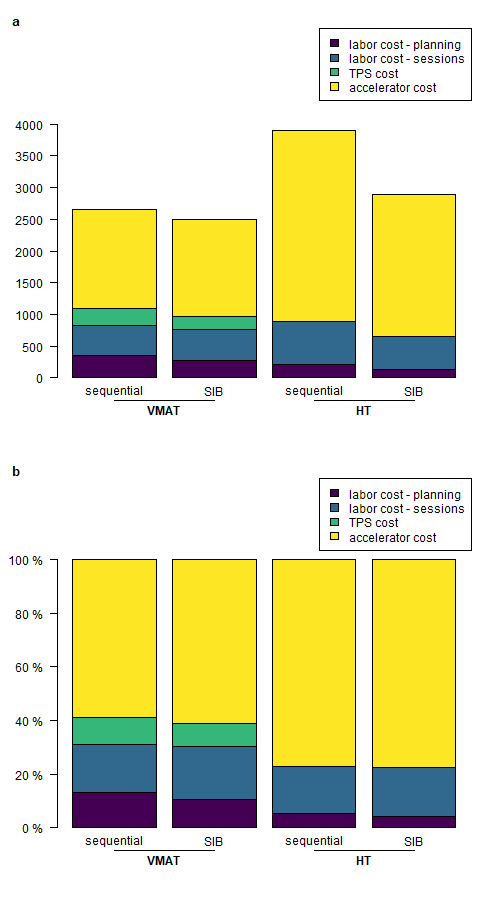


**Figure A3.** Barplots of the mean cost per patient according to the treatment plan (sequential/SIB): absolute costs (a) and relative costs (b).

Presented results are the results after IPTW.

Abbreviations: SIB = Simultaneous integrated boost, IPTW = inverse probability of treatment weighting, HT = helical tomotherapy
